# Supplementary material for: Acceptance and compliance with micronutrient powder (MNP) among children aged 6–23 months in northern Nigeria
Source: PLOS Glob Public Health. 2022 Oct 17;2(10):e0000961. doi: 10.1371/journal.pgph.0000961 (PMC10022258; doi:10.1371/journal.pgph.0000961)
Supplement: S1 Dataset — (ZIP) [file pgph.0000961.s008.zip › S8_Dataset/Spot Checks.zip!/Spot Checks/070715_A_GA_BA_2_SC.pdf]

# SPOT CHECK RECORDING FORM

**State: _ADAMAWA__ LGA: ___GANYE______ Community: ___GANYE 1 WARD____**

**Sex of index child: __MALE_____ Age of index child (in months): ___8 MONTHS______**

## Spot Check Information

1. **Date of MNP distribution to household** (circle option below based on LGA name above)**:**

   - **Kebbi:** BirninKebbi&Jega LGA: **June 16**
   - **Kebbi:** Danko-wasagu: **June 17**
   - **Adamawa:**Song, Ganye: **June 18**
   - **Adamawa:**Guyuk **June 19**

2. **Date of Spot Check Observation: ___07/07/2015________________**

3. **Number of sachets remaining**(Ask the caregiver to show you and count the specific number of sachets remaining)**:**

   12

4. **Number of sachets that should remainusing one MNP sachet per day** (Analyst only)**:**

   42

5. **Number of sachets remaining / number of sachets should be remaining**(Analyst only)**:**

   **____12_____ / ____42_____ = ____28.6_____ %**

## **Probes**

**Question 1.** You have **XX** MNP sachets remaining. Explain how easy or difficult it was to use the MNP product on a daily basis for your child 6 – 23 months? (Circle one answer)

| | | | |
|---|---|---|---|
| **Very Difficult** | **Difficult** | **Easy** | **Very Easy** |

**Question 2.**Describe any specific challenges that you faced that made the MNP difficult to use on a daily basis?

The challenge I faced using the product is due to the MNP powder not mixing well with the cerelac at the onset. But now, I've found a good formular which is pouring the MNP powder gradually while stirring

**Question 3.** Describe specific aspects of the MNP product that you or your child enjoyed that made it easier to use on a daily basis?

The MNP powder is to be added to foods like cerelac which is the nature of food the child accepts. Had it been it's to be added to what the child rejects or hardly accepts, it would have been a major challenge.

**Question 4.** What questions do you have about the MNP based on your experience using it?

i. Should mothers feed on the MNP and pass it through breast milk to the child?

ii. Let's say I travelled and spent somedays without giving the child MNP, is it advisableto continue the MNP when I returned back?

iii. Let's say I have 3 neighbours and I'm the only person that received the MNP for my child. After explaining to them on the importance of the MNP to the child while they saw me feeding my child with the product, is it advisable to share with them if they request? Because if I resist to share with them, they will feel offended. It will look as if I don't care for their own children as well and I'm being selfish. That may bring enmity between us which I dislike.

**Question 5.** What specific recommendations do you have that we can do as a program to make the MNP easier to use on a daily basis?

A pamphlet should be placed inside the sachet to indicate how the MNP works, the positive and negative signs to look after especially diarrhoea which I observed in the child when I started giving him the MNP. Had it been we knew that diarrhoea is one of the expected side effects of the drug, we won't have panic the way we did. If not for my husband and doctor that gave me the courage to continue the usage, I would have stopped using it.
Also, large number of caregivers should be reached to discourage sharing because the level of awareness about the MNP is high now and my neighbours are beginning to ask me questions on what is special about the product that I keep receiving visitors about and I'm not willing to share with them.
